# Developed and Validated Capillary Isotachophoresis Method for the Rapid Determining Organic Acids in Children’s Saliva

**DOI:** 10.3390/molecules28031092

**Published:** 2023-01-21

**Authors:** Justyna Dobrowolska-Iwanek, Małgorzata Jamka-Kasprzyk, Marcelina Rusin, Paweł Paśko, Sviatoslav Grekh, Anna Jurczak

**Affiliations:** 1 Department of Food Chemistry and Nutrition, Jagiellonian University Medical College, 31-008 Krakow, Poland; 2 Department of Pediatric Dentistry, Institute of Dentistry, Jagiellonian University Medical College, 31-155 Krakow, Poland; 3 Department of Analytical Chemistry, Faculty of Chemistry, Jagiellonian University, 30-387 Krakow, Poland; 4 Doctoral School of Exact and Natural Sciences, Jagiellonian University, 30-348 Krakow, Poland

**Keywords:** saliva, short-chain organic acids, children, isotachophoresis

## Abstract

One of the current challenges facing researchers is the search for alternative biological material, as opposed to routinely and invasively collected (such as blood), as the analysis of the former would provide information about the state of human health, allowing for the diagnosis of diseases in their early stages. With the search for disease biomarkers in alternative materials, the development of newer analytical solutions has been observed. This study aims to develop a reliable analytical method using the capillary isotachophoresis technique for the determination of organic acids in children’s saliva, the presence/elevation of which can be used in the future for diagnostic purposes. Organic acids such as formic, lactic, acetic, propionic, and butyric acid, were determined in the saliva of healthy children without carious lesions. The limit of quantification determined in the validation process was found to vary from 0.05 to 1.56 mg/L, the recoveries at the two levels were determined to vary between 90% and 110% for level I, while for level II the corresponding values of 75% and 106% were found; the presentation, expressed as relative standard deviation values (RSD), did not exceed 5%. The parameters determined while validating the results method indicated that the obtained are reliable. The Red–Green–Blue (RGB) additive color model was used for the evaluation of the method. This comparative analysis allowed us to define the color of the method, which expresses whether it meets the given assumptions and requirements. According to the RGB model, the isotachophoresis method developed requires less reagent input, shorter sample preparation times, and results with lower energy consumption. Thus, the subject procedure may provide an alternative, routine tool for determining organic acids in human saliva, to be applied in the diagnosing of diseases of various etiological origins.

## 1. Introduction

Saliva, which is an easily and noninvasively accessible body fluid, has been extensively studied as a potential diagnostic tool. It seems to be especially suitable in diagnosing children, as their suffering and traumatic experiences can be significantly reduced when compared to other procedures, e.g., blood collection. Saliva can be used to diagnose various autoimmune diseases, cardiovascular diseases, diabetes, human immunodeficiency virus (HIV), but also oral cancer, caries, and periodontal diseases. Numerous advantages, such as low cost, availability of home collection, or minimal risks of cross-contamination, confirm that saliva can be an alternative diagnostic material [1]. Our previous research with patients with inflammatory bowel disease indicated the importance of short-chain fatty acids (SCFAs) and lactic acid as useful biomarkers of the disease [2]. This prompts us to adapt this approach for diagnosing oral and periodontal diseases in children, based on the evaluation of the level of these acids in their saliva. Caries is a primary disease, the diagnosis of which the qualitative and quantitative analysis of SCFAs in saliva can be used in, as the demineralization of the tooth surface is associated with the production of these acids by cariogenic bacteria (i.e., *Leptotrichia shahii*, *Prevotella melaninogenica*, *Veillonella dispar*, *Streptococcus mutans*, *Scardovia wiggsiae*, *Parascardovia denticolens*, and *Lactobacillus salivarius*). It should be highlighted that none of the available salivary tests have shown reliable accuracy in detecting caries, thus the search for more novel ideas for such tests is still needed [3,4]. Periodontal diseases are characterized by the destruction of the tissues, such as gingiva and bone, combined with the activation of inflammatory mediators in response to periodontal pathogens and their products [5]. 

The analysis of SCFAs levels in saliva can also be used as a valuable tool in oral cancer diagnosis, as some positive correlations between several biomarkers in blood serum and saliva in patients with breast and lung cancer were recently observed [6].

There are three main sources of organic acids in saliva, namely the food consumed, the microorganisms inhabiting the plaque (as mentioned above), and finally the pure saliva components. A rapid increase in organic acid concentrations in the saliva is observed immediately after eating a meal, followed by a gradual decrease within approximately two hours following consumption, which determines the proper timing and the method of collecting saliva samples. Research shows that some acids, for example lactic, acetic, or formic acid, are formed as pyruvic acid fermentation products under the influence of microorganisms that inhabit the oral cavity environment [7,8]. The chemical profile of saliva collected with different procedures is the same, but the concentration of individual components can differ between specific samples, which is crucial to using the material as a potential biomarker of different diseases. Researchers have observed that saliva, collected after the stimulation of salivary glands, is more suitable for the assays for diagnostic purposes as it is less contaminated with food debris and microorganisms [9]. Non-stimulated collection of saliva, including passive salivation and draining, or saliva collection in the mouth and spitting out [10], provides inhomogeneous samples, i.e., requiring subsequent preparation steps. Apart from the collection method, other factors such as age, flow rate, diet, temperature, or pH influence both saliva secretion and composition, and thus establishing a standard for saliva collection is essential to obtaining reliable results [11].

Capillary isotachophoresis is classified as an analytical electromigration technique, in which the movement of the analytes to be determined in an externally applied electric field is directly proportional to the field intensity. In contrast to capillary electrophoresis, capillary isotachophoresis separation is based on the use of two different buffer systems with the sample placed between them, while in the former method a single basic electrolyte is used to fill the entire analytical system [12]. A recently published review, describing the applicability of this method in different aspects of biomedical analysis, emphasized the research potential of capillary isotachophoresis [13]. However, the method had not been used so far for the determination of SCFAs in saliva, the analysis of which was performed by ion chromatography [8], ultra-performance liquid chromatography (UPLC) [14], high-performance liquid chromatography (HPLC) [15], capillary electrophoresis (CE) [16] or nuclear magnetic resonance spectroscopy (NMR spectroscopy) [17] methods. Therefore, the objective of the study was to develop and validate the isotachophoresis method as being suitable for the determination of the selected SCFAs in the saliva of fasting children. We decided to focus mainly on formic, lactic, acetic, propionic, and butyric acids, all of which are either positively or negatively associated with oral diseases, as reported in the literature. The developed research procedure, which uses the isotachophoresis technique (ITP), may become a new tool in the future for saliva investigation for diagnostic purposes, where organic acids (their presence/concentration level) will serve as biomarkers for diseases of different aetiologies e.g., oral diseases. The additional aim of this study focused on the critical evaluation of the method in terms of analytical performance, safety and eco-friendliness, and productivity/practical effectiveness, to verify its suitability for SCFAs analysis, in comparison to other analytical procedures.

## 2. Results and Discussion

### 2.1. Separation of Organic and Inorganic Acid Ions

**Figure 1:**
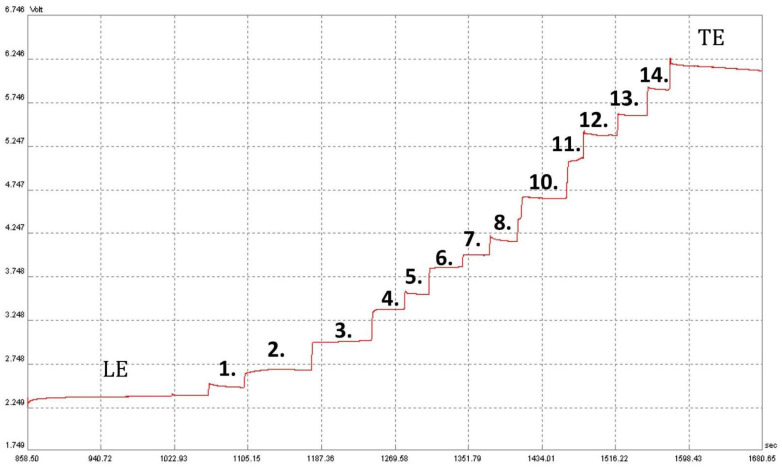
Isotachophoretic separation of acids in a standard mixture of fourteen acid anions (isotachophoregram). Zone assignments: LE—leading electrolyte; 1—nitric acid; 2—thiocyanic acid; 3—oxalic acid; 4—formic acid; 5—pyruvic acid; 6—citric acid; 7—phosphoric acid; 8—lactic acid; 9—succinic acid; 10—acetic acid; 11—ascorbic acid; 12—propionic acid; 13—butyric acid; 14—valeric acid; TE—terminating electrolyte.

Initially, the apparatus parameters, as well as the composition of the leading buffers (pH = 4.5) and terminating buffers, were selected to separate the potential acids present in saliva; the previously published studies facilitated appropriate selection. The pH value of the system was chosen to make the differences in effective ion mobilities large enough for their easy separation. Effective ion mobilities depend on (i) the degree of dissociation, which in turn depends on the pK value, temperature and pH in the zones, (ii) the correction factor for decreasing mobility of the relaxation and electrophoretic effects, which depends mainly on the ion concentrations, (iii) other factors such as solvation, ion radius and charge, as well as the dielectric constant and viscosity of the solvents [18]. In the present study the applied buffer composed of: (i) the leading electrolyte (pH = 4.5), which was a hydrochloric acid solution (10 mM), including 0.1% methylhydroxyethylcellulose (M-HEC) and a solution of epsilon-aminocaproic acid (20 mM); (ii) the terminating electrolyte, which contained 5 mM caproic acid and 5 mM of histidine, allowed us to separate inorganic and organic ions present in the saliva. In the leading electrolyte, the mobility of the separated ions of the individual compounds was the most diverse. It was possible to select the pH of the leading solution on the basis of tabular data from which one can read parameters describing ion mobility [19], depending on the pH of the environment and taking into account the selection of the appropriate acidity of the environment which enables the dissociation of studied compounds, vital in any electromigration method. A terminating solution is a compound whose ions have the lowest mobility in the whole system (among ions of the compounds to be determined) and hence caproic acid was chosen. A weak base, histidine, was added to increase its dissociation. Such an ITP separation system provides a stable and repeatable analytical signal namely, the conductivity of acid zones is stable at the given pH. Figure 1 shows an isotachophoregram, illustrating the separation of fourteen acid anions with a concentration of 6.25 mg/L.

### 2.2. Identification of Organic and Inorganic Acid Ions

**Figure 2:**
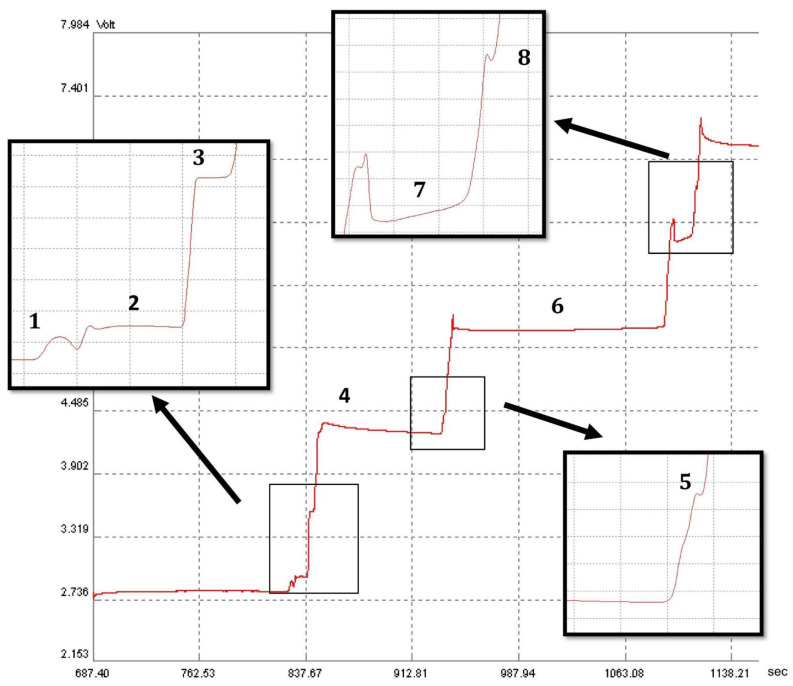
Isotachophoretic separation of acids in saliva (isotachophoregram). Zone assignments: LE—leading electrolyte; 1—nitric acid; 2—thiocyanic acid; 3—formic acid; 4—phosphoric acid; 5—lactic acid; 6—acetic acid; 7—propionic acid; 8—butyric acid; TE—terminating electrolyte.

The next step involved identifying the acids in the saliva collected from fasting children. For the correct identification, the acquired zones in the ITP analysis were determined by comparing the step heights for the standard solution containing pure compounds (SCFAs) and for the spiked samples. During the analysis, eight SCFAs were identified in the examined saliva samples, these being presented in Figure 2.

### 2.3. Validation of the Method

#### 2.3.1. Limit of Detection and Quantification, Calibration Curves

Linearity range, quantification limits, precision and recovery parameters were evaluated to validate the method. The calculated parameters are listed in Table 1. The limits of detection (LOD) for each analyte were determined experimentally. The prepared mixture of the standard solutions of 6.25 mg/L was diluted several times until its analytical signal could be reliably distinguished from zero; the lowest concentration of the corresponding acid was considered to be the limit of detection (see Table 1). The limit of quantification (LOQ) was three times the detection limit and was set as the first point of the calibration curve. The linearity range was assessed by plotting the zones’ length corresponding to each analyte against the acids’ concentration; the least-squares method was applied to calculate the respective correlation coefficients (R). According to the validation protocol, the determination coefficient met the criterion of R^2^  >  0.995; it was higher than 0.995 for all compounds. The method was characterized by the linearity of the indications in the following concentration ranges: 0.05–150 mg/L for acetic acid, 0.39–50 mg/L for formic acid, 0.39–100 mg/L for propionic acid, 0.78–50 mg/L for lactic acid, 0.78–50 mg/L for phosphoric acid, and 1.56–50 mg/L for butyric acid. 

#### 2.3.2. Precision and Recovery 

The precision of the method, expressed as the relative standard deviation value RSD, was established by successive analysis of sample solutions, with six independent repetitions for each, on the same working day. The RSD obtained ranged within 1.5–5.3%, which complied with the precision validation criterion for biological samples. Recovery was determined by spiking the sample with known concentrations of analytes at two concentration levels. Depending on the concentration of the analyte in the saliva, it was decided to adjust the amount of the acid added to maintain the total concentration within the calibration curve. Thus, for acetic and phosphoric acids, the addition of 25% and 50%, respectively, was applied, while for other concerned acids, 100% and 150% addiction were applied. The sample, which was prepared as described above, was subjected to three consecutive measurements. The recovery results, given in Table 1, were found to be satisfactory.

### 2.4. Organic Acid in Children’s Saliva Determined by Means of Other Analytical Methods

Few studies have been reported so far on the determination of SCFAs in children’s saliva. Some of these have focused on finding the differences in the metabolome of children with healthy dentition and children struggling with caries. Schultz et al. [14] studied the metabolites in the saliva and its biofilm of children (*n* = 57, aged 4 to 6) of different dental states in terms of caries activity. Saliva samples were taken between 9.30 a.m. and 10 a.m. in the dental clinic from children whose teeth we brushed without toothpaste and flossed about 30 min before the visit. The children were not allowed to eat or drink before the collection. The stimulated saliva was collected under medical supervision by spitting into sterile tubes. The saliva samples were cleaned of mucins and bacterial residues by centrifugation and proteins by precipitation with ice-cooled acetonitrile. In the next step, the saliva samples, enriched with the stable isotope-labelled internal standards (IS), underwent the derivatization process of organic acids and were subjected to analysis with the ultra-performance liquid chromatography/tandem mass spectrometry method (UPLC-MS/MS). As for the organic acids determined, acetic acid (357–2439.33 nmol/mL), propanoic acid (33–1295 nmol/mL) and lactic acid (1.5–386 nmol/mL) showed the highest concentrations. Formic acid was not determined. No significant differences were observed in the saliva or pellicle in relation to caries activity, e.g., no differences in the mean concentrations of determined organic acids were recorded. Fidalgo et al. [17] reached different conclusions. Sixty-five systemically healthy children attending the dental clinic were recruited for the study. Saliva from children at all stages of dental development, from the appearance of a set of deciduous teeth to the last permanent dentition, was analyzed. The study group included both children with healthy dentition and those with dental caries. Patients (around 10 a.m.) passed 3 mL of unstimulated saliva by expectoration for approximately 5 min. Two hours before the collection, they were asked to refrain from oral activities. Saliva samples were analyzed using NMR spectroscopy. The saliva of children with carious lesions was characterized by higher concentrations of lactate, acetate, and n-butane compared to the control group. On the contrary, Ahluwalia et al. [15] studied the saliva of children aged 6 to 12 years with cleft palate (*n* = 81 and control group—*n* = 61). When saliva was collected, the children were asked to sit with their heads tilted forward and drool in a sterile container; no stimulation was applied. Approximately 2 mL of saliva was collected from each subject. Before the proper analysis, all saliva samples were purified by being passed through a C_18_ cartridge and then analyzed using the HPLC technique. The analysis yielded the following ranges of acid concentrations: 19 ± 1–30 ± 8 µM for formic acid, 43 ± 3–63 ± 7 µM for acetic acid, and 127 ± 19–180 ± 19 µM lactic acid; succinic acid was not determined in the work. Statistical analysis of the results obtained indicated that the concentrations of acetic, formic, and succinic acids were significantly higher in the saliva of the children from the control group compared to those with cleft palate (*p* < 0.04). For lactic acid, no such relationship was found. 

The studies presented indicate that different analytical techniques are used to analyze children’s saliva for the determination of organic acids, with different apparatus systems, often very costly, also involved in their operation. In addition, the sample preparation procedure for the measurement is often a complex, multistep process, requiring the use of additional reagents. Therefore, the analytical procedure developed for the determination of organic acids in juvenile saliva using capillary isotachophoresis may become a good alternative.

### 2.5. Application of the RGB Model for Method Evaluation

An important aspect of the development of a new analytical method is its critical evaluation, and a useful tool for this purpose is the RGB additive color model [20]. This model was used to compare the developed isotachophoresis method with the previously developed UPLC-MS/MS method selected by Schulz et al. [14]. This comparative analysis is based on three aspects of analytical methods: analytical performance, safety and eco-friendliness, and productivity/practical effectiveness. Therefore, when building the model, the following aspects were taken into consideration: the amount and toxicity of chemicals, and the costs associated with the validation and analysis of compared analytes, such as components of the mobile and stationary phases and buffer, internal standards and standards of organic acids, and also other reagents necessary for the calibration and sample preparation. The process of collecting and preparing saliva samples, analysis time, as well as energy consumption, was also taken into account (based on Nowak et al. [21]). The comparison of the methods evaluated is presented briefly in Table 2. Further details including, methodology and assumptions, were included within the electronic Supplementary Material (ESM).

The final color of the isotachophoresis method is white, which means that this method is a good candidate for this application. However, the final color for the UPLC-MS/MS method is grey, which means that this method can be considered if no better methods are available [20]. The lower value of the “Method brilliance” parameter for the UPLC-MS/MS method results from a greater number of reagents used, and thus from the number of pictograms and the cost of these reagents. This is related to the use of the isotope-labelled reagents as ISs and the derivatization of the analytes carried out before the chromatographic analysis. Whereas, the isotachophoresis method is characterized by much better practical effectiveness, which is especially due to the short time taken to collect and prepare saliva samples for analysis. Because of this, it can be seen that the developed isotachophoresis method, although less automated in the case of this particular instrumental setup, requires less reagent input, shorter sample preparation times, and results in lower energy consumption. These factors lead to a fast and reliable method for the determination of organic acids in saliva.

## 3. Materials and Methods

### 3.1. Sample Collection and Sample Preparation

Due to the age group of children involved in the present experiment, it was decided to sample fasting saliva, which was stimulated by chewing a cotton swab. This procedure allowed the sampling process conditions to be reproducible with respect to similar sampling time, same body position, same chewing time and manner, etc. This noninvasive method is comfortable for patients, and the amount of saliva collected was large enough to carry out all the planned experiments, with repeated measurements.

Saliva samples were collected at the fasting state of children (*n* = 3). The patients were three girls, two of whom were twelve years old, and one seven. The children were generally healthy, with no burden of systemic diseases. On the day of examination, no symptoms of systemic infection were observed. Carious or inflammatory lesions were found, although no signs of periodontopathic disorders were detected. The result of the ICDAS (International Caries Detection and Assessment System) test was 0, which corresponds to healthy dentition. Parents were asked to sign a consent form for the examination and the children were prepared for it. Patients were asked not to eat meals or consume any liquids before the examination. Saliva samples were collected between 8 and 10 a.m. with subjects chewing a cotton swab without any saliva-stimulating agent for 5 min to avoid interference. The patients sat comfortably with their eyes open and their heads slightly tilted forward to facilitate saliva collection from the floor of the mouth. The swab was then placed in a sterile plastic centrifuge tube provided by the manufacturer (Salivette Sarstedt Nuembrecht Germany System). Material preparation for the examination started no later than 2 h after sampling. The saliva samples were centrifuged for 10 min at 900 rpm at 4 °C, and the resulting filtrate was transferred to a sterile 2.0 mL microtube. The samples were frozen until analysis at −21 °C. Before analysis, the samples were thawed, diluted 3.5 times with distilled water, and filtered using 0.45 µm nylon filters (La-Pha-Pack, Langerwehe, Germany). The study protocol was approved by the Bioethics Committee at Jagiellonian University Medical College in Kraków, Poland (1072.6120.106.2019; as of 27 June 2019). To protect the sample from bacterial contamination and proliferation of microorganisms present in the sample, saliva collection and subsequent handling (centrifugation, dilution, or filtration) were carried out according to the relevant recommendations and requirements of the principles of aseptic rules. In addition, the utmost care was taken to ensure that the time from sample collection from patients to freezing was as short as possible. Sample centrifugation, which was the longest step in sample preparation for measurement (10 min), was carried out at 4 °C. The analytes discovered in biological samples were found to be resistant to temperature changes associated with the freezing and thawing of samples as well as those occurring during measurement at room temperature (results not shown).

### 3.2. Reagents

The organic acids used were of analytical standard grade. Caproic and valeric acids were obtained from Fluka (Steinheim, Germany); succinic acid from Lancaster (Morecambe, UK); 95% formic, thiocyanic, nitric, pyruvic and ascorbic acids, as well as monohydrate citric acid, plus oxalic, propionic, butyric, 85% ortho-phosphoric acids from Sigma-Aldrich (Saint-Quentin-Fallavier, France) and 80% D,L-lactic acid from Avantor Performance Materials Poland S.A. (Gliwice, Poland). A 36–38% hydrochloric acid was delivered by Baker Analyzed and methylhydroxyethylcellulose (M-HEC) by HERCULES (Prague, Czech Republic), β-alanine by Merck (Darmstadt, Germany) and L-histidine by Serva (Heidelberg, Germany); 99.5% acetic acid was obtained from Chempur (Piekary Śląskie, Poland). Deionized water of 18 MΩcm was obtained from Milli Ro & Q water purification system (Merck-Millipore, Billerica, MA, USA).

### 3.3. Instrumentation

Isotachophoretic separations were performed using the Electrophoretic Analyser EA 202 M (Villa Labeco, Spisska Nova Ves, Slovakia) with the conductivity detector. The system was equipped with a sample valve of 30 μL at a fixed volume and two capillaries, namely the pre-separation capillary (160 mm × 800 µm I.D.), and the analytical capillary (160 mm × 300 µm I.D.). The pre-separation involved a current of 250 μA, while the actual separation in the analytical capillary column was performed at 60 μA during the initialization phase and at 50 μA during the detection phase. The following solution was used as the leading one (pH = 4.5): hydrochloric acid solution (10 mM), including 0.1% methylhydroxyethylcellulose (M-HEC) and epsilon-aminocaproic acid solution (20 mM). The terminating electrolyte contained 5 mM of caproic acid and 5 mM of histidine. Analysis time per sample did not exceed 20 min.

## 4. Conclusions

Children’s saliva can provide an alternative test material, with noninvasive sampling that does not cause stress to the minors. It is worth noting that the production of saliva and its composition changes with age, as well as under the influence of drugs/abuse and/or disease, and hence the choice of study saliva composition at this age group seemed more beneficial. The analytical method described in this article provides a reliable determination of five organic acids in children’s saliva. The validation parameters indicated that the method could provide a tool for determining salivary acid concentrations. The monitoring of the concentrations of these analytes may also serve in the future as part of a study of the progression or treatment process of the disease. Additionally, saliva sample preparation for SCFAs measurement using isotachophoresis is quick and simple, with the only technicalities requiring for the saliva sample to be diluted with water and filtered. The analysis also consumes a small amount of the reagents; thus, the amount of waste is strongly reduced. Moreover, reagents of low toxicity are used, which is in line with the green chemistry trend.

## Figures and Tables

**Table 1:** Validation parameters for organic acids in saliva samples obtained using the isotachophoresis method.

Acids	Conc. (mg/L)	LOD (mg/L /µM)	LOQ (mg/L /µM)	R^2^	Recovery (%) Level I	Recovery (%) Level II	RSD (%) (*n* = 6)
formic acid	7.5 ± 0.3	0.13/2.82	0.39/8.47	0.9997	107	97	5.0
lactic acid	6.0 ± 0.3	0.26/2.65	0.78/7.95	0.9997	90	75	4.9
acetic acid	502 ± 8	0.02/0.28	0.05/0.83	0.9988	97	106	1.5
propionic acid	64 ± 2	0.13/1.76	0.39/5.27	0.9961	110	105	3.2
butyric acid	5.7 ± 0.1	0.52/5.68	1.56/17.04	0.9956	100	101	1.9

**Table 2:** Comparison of evaluation of the analytical methods for the determination of organic acids in children saliva.

Method	Final Color	Redness	Greenness	Blueness	Brilliance (MB)
Isotachophoresis	White	85.1%	68.6%	81.8%	78.2%
UPLC-MS/MS	Gray (colorless)	59.2%	49.3%	53.5%	53.8%

## Data Availability

The data presented in this study are available on request from the corresponding author

## Supplementary Materials

The following supporting information can be downloaded at: https://www.mdpi.com/article/10.3390/molecules28031092/s1, The Excel worksheet: Parameters considered in the construction of the RGB model and details of its design.

## Limitations

Our study has its limitations. It should be concerned solely as a pilot study with a small number of healthy female participants (*n* = 3), and saliva collection was performed using one type of method.
